# From Primary Cell to Organoid: the proliferative fate international conference and workshop

**DOI:** 10.1242/bio.061970

**Published:** 2025-07-18

**Authors:** Mohamed Jemaà, Amine Bourzam, Marwen Ben Fdilen, Mariem Sellami

**Affiliations:** ^1^ Human Genetics Laboratory LR99ES10, Faculty of Medicine of Tunis, Tunis El Manar University, Tunis 1006, Tunisia; ^2^ Neurophysiology, Cellular Physiopathology and Valorisation of Biomolecules Laboratory LR18ES03, Faculty of Sciences of Tunis, Tunis El Manar University, Tunis 2092, Tunisia; ^3^ Department of Biology, Faculty of Sciences of Tunis, Tunis El Manar University, Tunis 2092, Tunisia

**Keywords:** Cell culture, Primary cells, 2D and 3D culture, Spheroids, Organoids, Hands-on workshop

## Abstract

Cell culture is an essential tool for basic, biomedical and translational research. This can be as routine as growing cell lines for toxicity assays, as specific and personalised as patient-derived cell culture, or it can mimic physiological conditions such as organoid culture. In order to train and promote collaborative research among North African researchers in the field of cell culture, a consortium formed by the Faculty of Sciences of Tunis, Tunisia, the Laboratory of Neurophysiology, Cellular Physiopathology and Valorisation of Biomolecules, the Merck Group and Learn and Win decided to organise a North African conference and workshop on complex cell culture, from primary cells to organoids. For 1 week, from 18 to 23 November 2024, more than 500 young researchers and students in the life sciences and more than 30 international speakers and trainers met at the Faculty of Sciences in Tunis, Tunisia, to discuss cell culture technology and protocols, from primary cell culture to organoids, with a strong focus on technologies and discoveries. This meeting review describes the scientific event and highlights the main results of both the conferences and the practical sessions.

## Teaser

**Summary:** A consortium formed by the Faculty of Sciences of Tunis, Tunisia, the Laboratory of Neurophysiology, Cellular Physiopathology and Valorisation of Biomolecules, the Merck Group and Learn and Win organised a North African conference and workshop on classical and complex cell-culture protocols.

## Why a conference on cell culture?

As cell biologists and cancer researchers, our work is fundamentally linked to cell culture, whatever the purpose of the assay, from the most basic to the most complex. There are a number of things that condition cell culture: a well-equipped cell culture room, of course, consumables, the type of cells we use and whether we use additional technologies such as microscopy or flow cytometry. In addition, research laboratories in low- and middle-income countries such as Tunisia and North Africa in general face additional difficulties and challenges when conducting cell culture. For example, equipment and materials are expensive relative to our local budget, and the bureaucratic procedure of the customs services blocks the shipment for days, causing the cells to die because of thawing. These technological needs and challenges force us to reorganise our benches and adapt our experiments, and it makes us more flexible in the way we deal with the assays.

For example, and because of the challenges we mentioned, protocols change and are adapted to the lab (when we need to add antibiotics and when not, for example, using a broad-spectrum medium such as DMEM instead of McCoy's 5A for colon cancer cells, etc.). With our team, we have shifted towards more use of primary cells, biopsies and use of different protocols depending on the tissue. Here, an example of secondary culture of astrocytes after rat brain extraction, (Bourzam et al., 2024) or another example of red blood cells erythrocytes for basic toxicology (Jemaa et al., 2023), or even Patient Derived Xenograft PDX (Simon Serrano et al., 2020).

More importantly, the use of primary cells culture, guarantee the genomic and phenotypic stability and the retention of key characteristics of the tissue, or disease related biopsies, especially in cancer research. *A contrario*, immortalised cells lines are subject to genetics changes after prolonged passage as a result of selective pressures, (just thinking about HeLa cells, established in the early 1950s – last century!) also these cells could be the host of invisible contamination, like mycoplasma, or even misidentified or cross-contaminated by other cells (Lorsch et al., 2014; Capes-Davis et al., 2010).

At the same time, to address more complex problems, such as metastasis, in a 3D microenvironment, we had to master *de facto* more complex cell culture methods and assays, such as spheroids, and start using organoids to address another budgetary and ethical problem, which is the use of animal models (Park et al., 2024) (the need to replace or at least reduce and refine the use of animals, known as the 3Rs principle (eBioMedicine, 2022).

Therefore, and to build a whole-process story about the need and management of cell culture with both technological and budget challenges, organising a master class with both conferences and workshops for North African researchers was a must. To host such an exciting scientific event, we chose the City of Science of Tunis (cst.rnu.tn/en) as the main venue for the conferences and the Faculty of Science of Tunis (fst.rnu.tn/en) as the venue for the workshops.

## What were the key themes of the meeting?

The conference started on Monday 18 November 2024 with an introduction to cell culture by Mohamed Jemaà from the Laboratory of Human Genetics, Faculty of Medicine, Tunis (FMT) and the Laboratory of Neurophysiology, Faculty of Sciences, Tunis (FST), Tunisia. The conferences emphasised the importance of cell culture in cell biology and related research, especially cancer research. The conference was followed by a talk entitled ‘Primary Neuronal and Neural Cell Culture’ by Olfa Masmoudi, Head of the Neurophysiology Laboratory at FST. The main message was how to reproduce synaptic networks of neurons in culture and how to use primary neuronal cultures to observe the formation of neurites, which are pseudo-axons that can develop and differentiate into dendrites. The talk also explored methods for isolating primary glial cells, such as astrocytes, or neurons from specific regions of nervous tissue in neonatal rats or mouse pups. Laura Senovilla, Principal Investigator of the ‘Cell Stress and Immunosurveillance’ team at the Instituto de Biología y Genética Molecular (IBGM) at the Faculty of Medicine in Valladolid, Spain, gave a talk entitled ‘Mammary Stem Cell Culture’ in which she discussed how to use mammary cells for primary culture. This included cells derived from human breast cancer tissue and human adipose-derived stem cells, which she used to illustrate the importance of primary cell culture. Raja Marrakchi, from the Immunology Laboratory at FST, took the lead in presenting a technical talk on ‘How to Establish a De Novo Cell Line’. This explored different techniques for immortalising cells, ranging from primary cultures to tumour cells and organoids, for use in pharmacological studies or to investigate the effects and mechanisms of action of various drugs. This was a continuation of the previous cell culture lectures.

Ines Limam, from the Laboratory of Human Genetics at the FMT, then gave a presentation entitled ‘Cell Culture Lab: From Media Preparation to Cryopreservation – Tips and Tricks’. Cryopreservation protocols involve preserving cells through a sequential freezing process from −80°C to −196°C. The presentation also provided tips for preventing contamination in cell culture and highlighted essential rules that researchers should follow in the cell culture laboratory.

Yasmine Jibry from the Merck Group then gave a presentation on ‘Cell Culture Lab Safety Practices’, covering the potential sources of contamination in cell culture, the various types of microbial contamination and the visual identification of microorganisms in cell culture, as well as the use of sterile filtration to prevent contamination. This was followed by Izzeddine Khanfari, also from the Merck Group, who gave a presentation on ‘The Vital Role of Water Quality in Cell Culture’, covering the use of optimal water in various cell culture applications and the purification methods used to obtain ultrapure water.

From the general context to concrete research in primary cells, Asma Chikhaoui, from the Laboratory of Biomedical Genomics and Oncogenetics at the Institut Pasteur de Tunis, gave a presentation entitled ‘Exploring the effect of nicotinamide supplementation during ageing in different cellular models from patients with Cockayne syndrome’, in which she demonstrated the regulatory effect of nicotinamide in promoting the regeneration and repair of damaged muscle tissue. Then, Mohamed Jemaà closed the primary cell session with a presentation on ‘Erythrocytes as a model cell in pharmacology’, demonstrating how erythrocyte cultures can be used for pharmacological studies and drug discovery, with an assessment of various biological pathways, including calcium transport, apoptosis and oxidative stress.

Day 2 began with a presentation on using cell culture to identify and validate therapeutic targets. Mohamed Abdelkarim, from the Laboratory of Human Genetics at FMT, gave a talk on ‘The pharmacological properties of plant extracts on cancer cells’, providing the example of the potential of green tea extract and epigallocatechin-3-gallate (EGCG) on breast cancer spheroids. Then Wassim Moslah from the Laboratory of Biomolecules, Venoms and Theranostic Applications at the Institut Pasteur of Tunis gave a talk on ‘The anti-glioblastoma effect of a scorpion venom‘. This involved the identification and isolation of biomolecules from the venom, followed by the design of multi-branched molecules targeting U87 cancer cells. Nesrine Kerkeni, from the Charles Nicolle Hospital in Tunis, Tunisia, provided additional information on glioblastoma in the form of a presentation entitled ‘Comparative Transcriptomic Analysis of U87 Glioblastoma Cells Treated with Various Therapeutic Agents’. This presentation showed that a combination of cell culture and in silico screening of diverse compounds targeting multiple signalling pathways may offer effective therapeutic strategies against glioblastoma. The session ended with Nouha Setti-Boubaker from the Department of Research, Diagnosis and Innovative Technologies at the IRCCS-Regina Elena National Cancer Institute in Rome, Italy. She presented her work on ‘CRISPR Cas editing in mammalian cancer cells’ to generate clones of non-transformed human cells carrying a collection of ATM variants. The aim is to study their phenotypes and assess their tumourigenic potential.

We moved forward in the complexity of cell culture and “From 2D culture to 3D culture models” was the title of Maram Morjan conference from the Laboratory of Biomolecules, Venoms and Theranostic Applications, Institute Pasteur of Tunis. She presented a 3D multicellular spheroid of hepatocellular carcinoma (HCC) and how to generate it. Ilio Vitale, PI of the Genomic Instability and Tumour Immunity team at the Italian Institute for Genomic Medicine, Candiolo, Torino, Italy, took the lead with a presentation entitled ‘Boosting genomic instability to overcome chemoresistance of and immune evasion by cancer stem cells’. He demonstrated that whole-genome duplication contributes to genetic diversity. Meanwhile, the immunogenicity of cancer stem cells *in vivo* influences tumour progression and their interaction with the immune system, including their susceptibility to immune-mediated attack. He used 3D cells culture and spheroid in his work. Christiaan F. Labuschagne from North-West University, South Africa, continued with a talk on ‘Tumour microenvironment in metastasis’ showing the effect of pH on cancer cell proliferation, adhesion, and migration, as well as its impact on cellular metabolism. Samer Abdallah from the Faculty of Medicine and Health Sciences, An-Najah National University, Nablus, Palestine took the lead and presented his work entitled ‘Regulation of epithelial cancer cell detachment and migration using multicellular tumour spheroid (MCTS) model’. He demonstrated how to inhibit cancer cell growth and migration, and explained the role of the MCTS model in understanding cancer progression and resistance, as well as in screening anti-cancer treatments. Davide Valente from the Institute of Molecular Biology and Pathology (IBPM), National Research Council of Italy (CNR), Italy gave a talk about the ‘3D Gastrointestinal Cultures: tools in cancer cell biology with a focus on Homeodomain Interacting Protein Kinase 2’. He provided a detailed description of HIPK2 and its role in cytokinesis and tumourigenesis. First, in 3D and organoid cultures, and then in a murine model of pancreatic carcinogenesis, HIPK2 depletion reduces pancreatic tumour formation in the genetically engineered KC mouse model. Wided Kelmemi from Merck group ended the session with presentation entitled ‘3D cell culture models to understand cancer biology’. She presented different types of 3D culture and the use of organoids to model the structure and function of organs, along with various products from Merck used to perform 3D cultures.

We started the session on patient-derived xenograft (PDX) models in complex cell culture models with a lecture on ‘PDX as preclinical neuroblastoma models’ by Daniel Bexell from the Division of Translational Cancer Research, Department of Laboratory Medicine at Lund University, Sweden. He discussed the role of PDX models in studying neuroblastoma and establishing a system that is predictive of clinical outcomes. This was followed by a presentation entitled ‘PDX in paediatrics’ by Adriana Mañas Núñez from the Paediatric Oncohaematology Group at IdiPAZ-CNIO in Madrid, Spain. PDX models are an ideal tool for overcoming the challenges of paediatric cancer research, such as the limited number of samples and the difficulty of conducting clinical trials for identifying biomarkers or screening drugs. Ioannis Sotiropoulos, from the ExoBrain Institute of Biosciences and Applications at the NCSR in, Athens, Greece, ended the session with a talk entitled ‘Exosomes as new “tools” for diagnosis and treatment in the new era of precision medicine’. He discussed the study of brain-derived exosomes as biomarkers and their use in treating brain damage, chronic stress, depression, and Alzheimer's disease pathologies.

On day three, also referred to as ‘Organoids Day‘, Sara Abdel Rehim Soliman from the Italian Institute for Genomic Medicine in Candiolo, Turin, Italy, gave a conference entitled ‘Exploring mitotic checkpoint functionality in patient-derived spheroids and organoids’. She discussed how mitotic checkpoint deregulation can be exploited using an automated method to identify vulnerabilities in cancer cells and discover novel drugs, with organoids serving as the main model. Then Isabel Palacios, from Queen Mary University of London, gave a talk entitled ‘Tissue mechanics in 3D human organoids’. She aimed to understand the morphogenetic processes of neuroepithelial cells within neural rosettes formed in cerebral organoids to better model early brain development and neurodevelopmental disorders. She was followed by Katja Röper, Principal Investigator at the MRC Laboratory of Molecular Biology at the University of Cambridge, UK, who presented ‘The human renal mesenchymal-to-epithelial transition’ and explained how renal organoids serve as a promising in vitro model to study human nephron development, offering insights into kidney morphogenesis, disease modelling and regenerative medicine. Samantha Nicholson, representing the Merck Group, gave a talk entitled ‘Optimising Organoid Culture: The Toolbox’, where she presented some of the company's new facilities for starting organoid culture.

Mohamed Jemaà concluded the conference with a presentation entitled: ‘From Primary Cells to Organoids: A Short Story’. He presented work that started with patient-derived cell culture, then the establishment of primary cell cultures and cell lines, and finally spheroids and organoids, ending with a small animal model for experimentation to propose a new treatment for cancer metastasis. The goal of the presentation was to summarise the philosophy of the organised conference, ‘From primary to organoids’, with a concrete research project.

In addition to the lectures and presentations, we also had poster sessions during the breaks, where young researchers presented their research using cell culture on various topics. The 3-day session was also fully accessible to participants (although pre-registration was mandatory). With around 400 registrations and 200 people in the amphitheatre each day, the event was very successful. The conference was very rich in terms of connection between attendees and speakers, with an exciting question and answer (Q&A) session. Recordings of the conferences are also available on YouTube. (https://www.youtube.com/playlist?list=PLXohrS5wWCq_g19_DabeD2PGSOmJs0cpL).

**Fig. 1.**
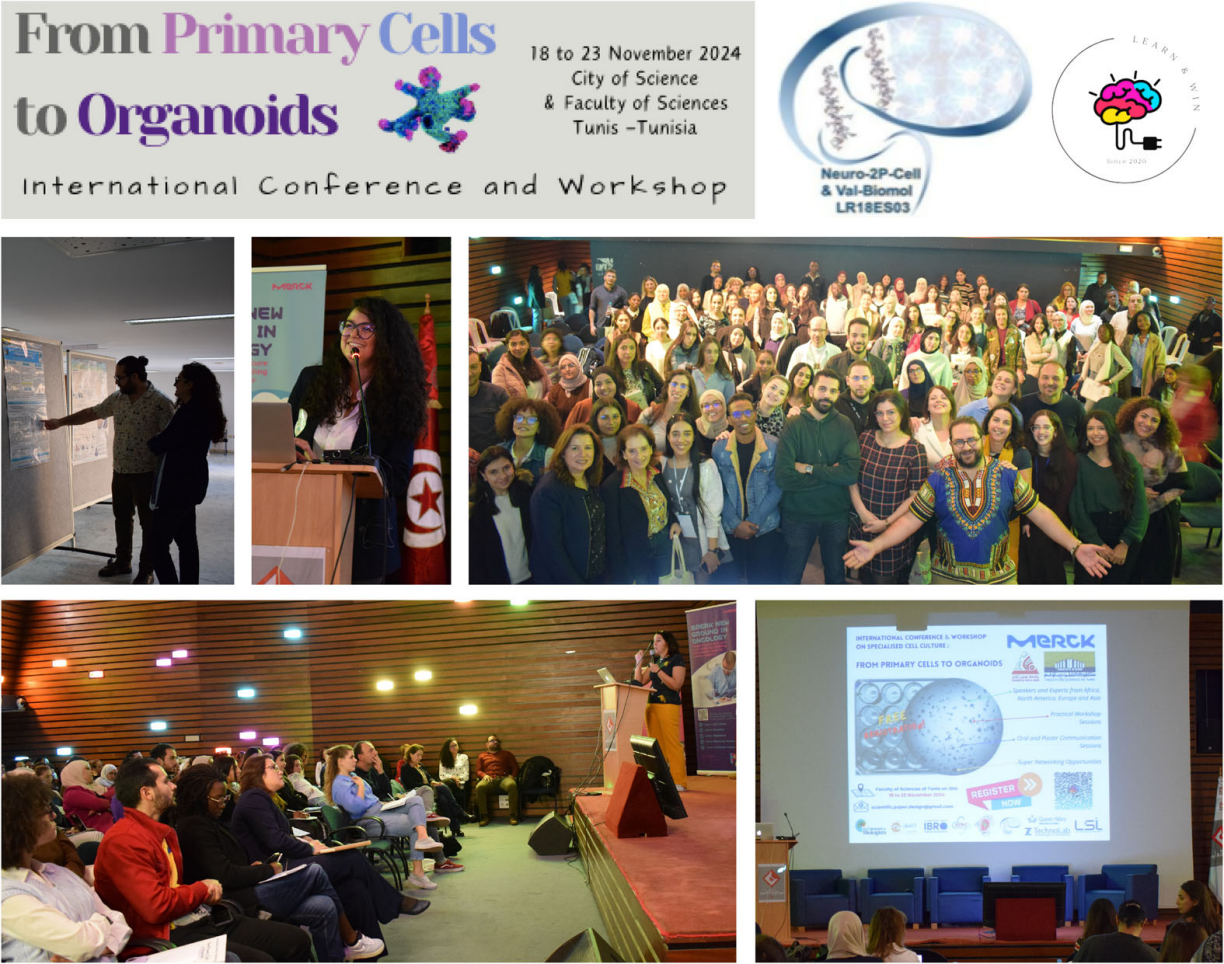
**Attendance overview for conferences and poster session**.

In addition, and at the end of each session (five sessions in total), we organised a quiz on what was presented via the kahoot application and we awarded five laureates and also gave two prizes for the best participants (young researchers who asked the most interesting questions to the speakers and also who visited and interacted with poster presenters during the poster session). All this made the conferences and the poster session a huge success and amazingly fun (Fig. 1).

## Workshop and practical sessions

**Fig. 2.**
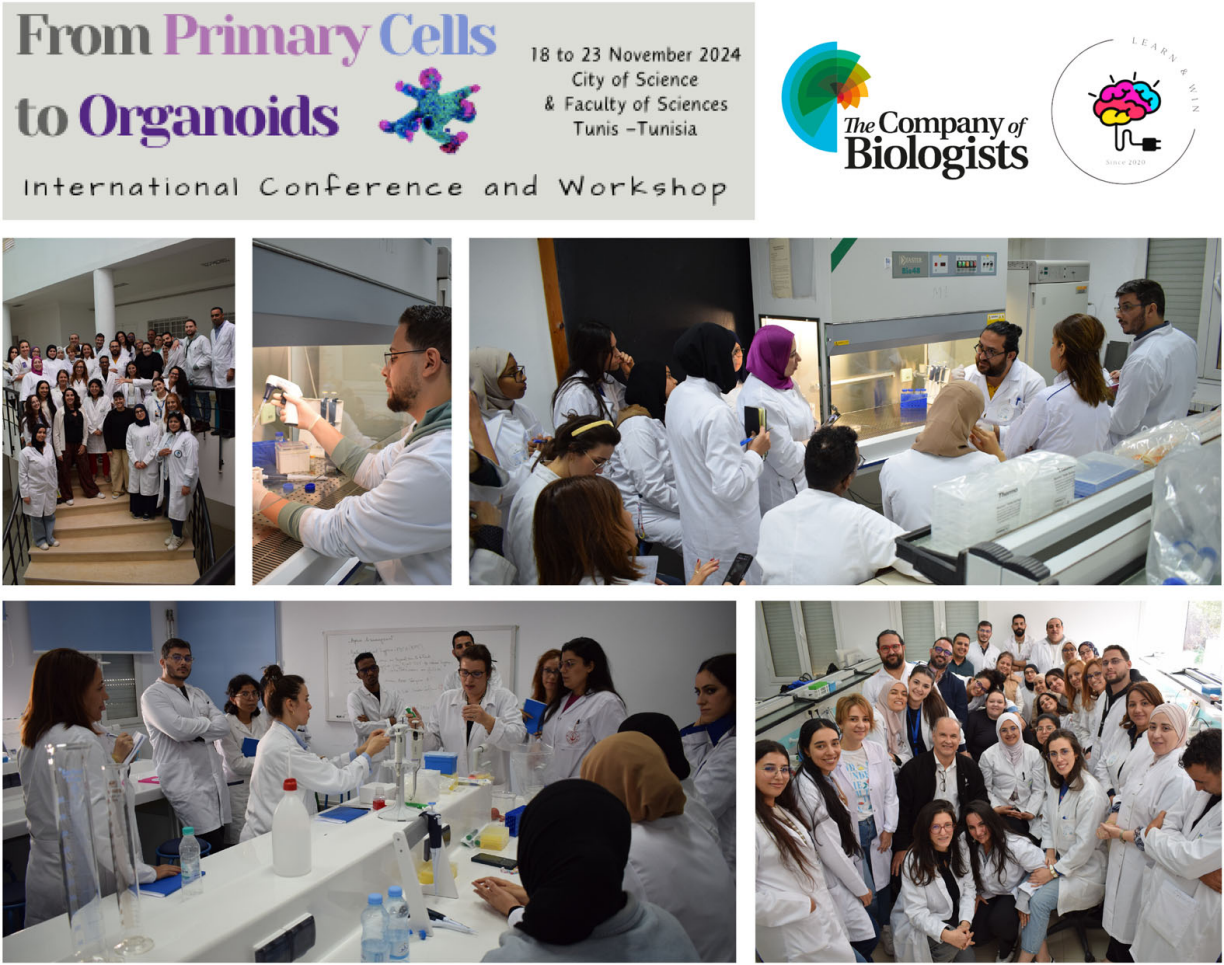
An overview of the practical sessions.

As a follow-up to the conference, we organised a 3-day workshop on cell culture, from primary cells to the establishment of organoids. Again, we received a large number of international applications and selected 20 participants, mainly PhD students, from Tunisia (13), Mauritania (1), Algeria (4), Egypt (1) and Italy (1). The workshop started on Thursday 21 November and took place at the Cell Biology Training Platform of the Department of Biology, Faculty of Sciences of Tunis, Tunisia. Following the same strategy, we started with the culture of primary cells. We first euthanized two mice from the animal facility of the Faculty of Sciences, then we dissected the animals to extract the brain, spleen, liver, ears and mammary glands to establish primary culture of neuron and glia cells, mouse adult fibroblasts (MAFs) from ears, and liver, spleen and mammary primary cells. To allow all participants to manipulate and perform the experiments themselves, and to run the workshop smoothly, we used two different labs in the platform, with three groups in each lab and one trainer for each group, in addition to a coordinator in each lab. The afternoon session was dedicated to classical cell culture, i.e. the entire process from harvesting and collecting adherent (pre-prepared) cells, counting, media preparation and all passaging protocols to cryopreservation (Fig. 2). The day ended with a Q&A session.

The second day was dedicated to 2D and 3D culture, from the preparation of special media and the coating of plates, or the use of a more specific material such as low-adhesion plates, to the creation of 3D spheroids. For each experiment, a pre-prepared culture was performed by the organising team for final observation using microscopes and a confocal microscope for stained cells. To give the participants extra tools, especially in the context of very specialised cell culture, we ran a session on media preparation and filtration for specific cells at the end of the day.

**Fig. 3.**
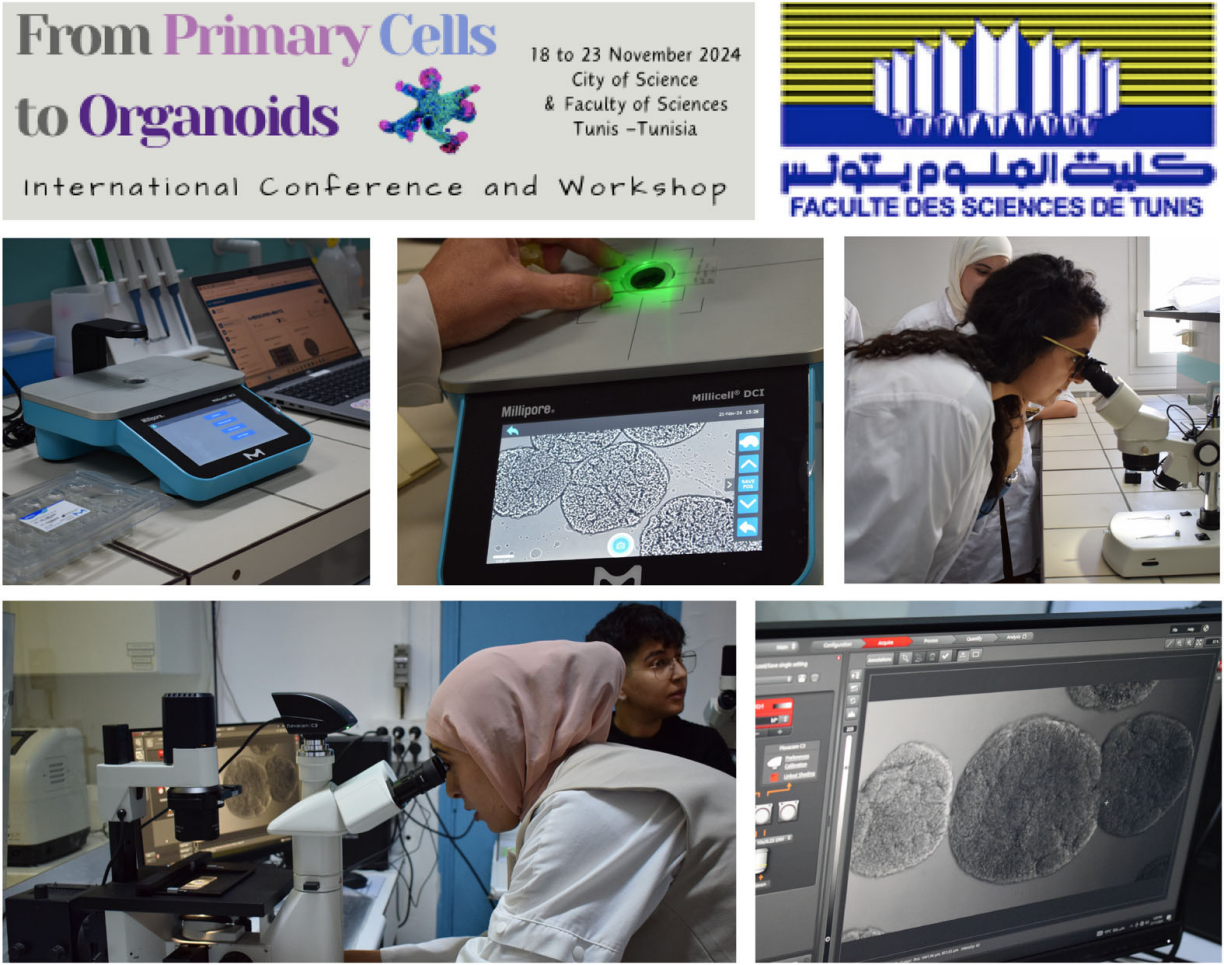
An overview of the practical sessions, focus on organoids.

Day 3 was all about organoids. We manipulated different types of organoids generously donated by the team of Katja Röper from Cambridge and Isabel Palacios from Queen Mary University of London. We spent time in the confocal microscopy room observing organoids with different stains, as this model is quite new to our participants and to North Africa in general (Fig. 3). We also made a small change to the programme and manipulated *Drosophila* fruit flies with cancer to introduce the next level of cell culture for small animal xenotransplantation. Brain organoid in *Drosophila* as a target in an ongoing collaborative project between the neurophysiology lab at FST and the lab of Isabel Palacios at Queen Mary London. The day ended with a longer question and answer session to summarise the whole workshop and answer any questions that people may have had that they did not have time to ask.

## Social events and networking

**Fig. 4.**
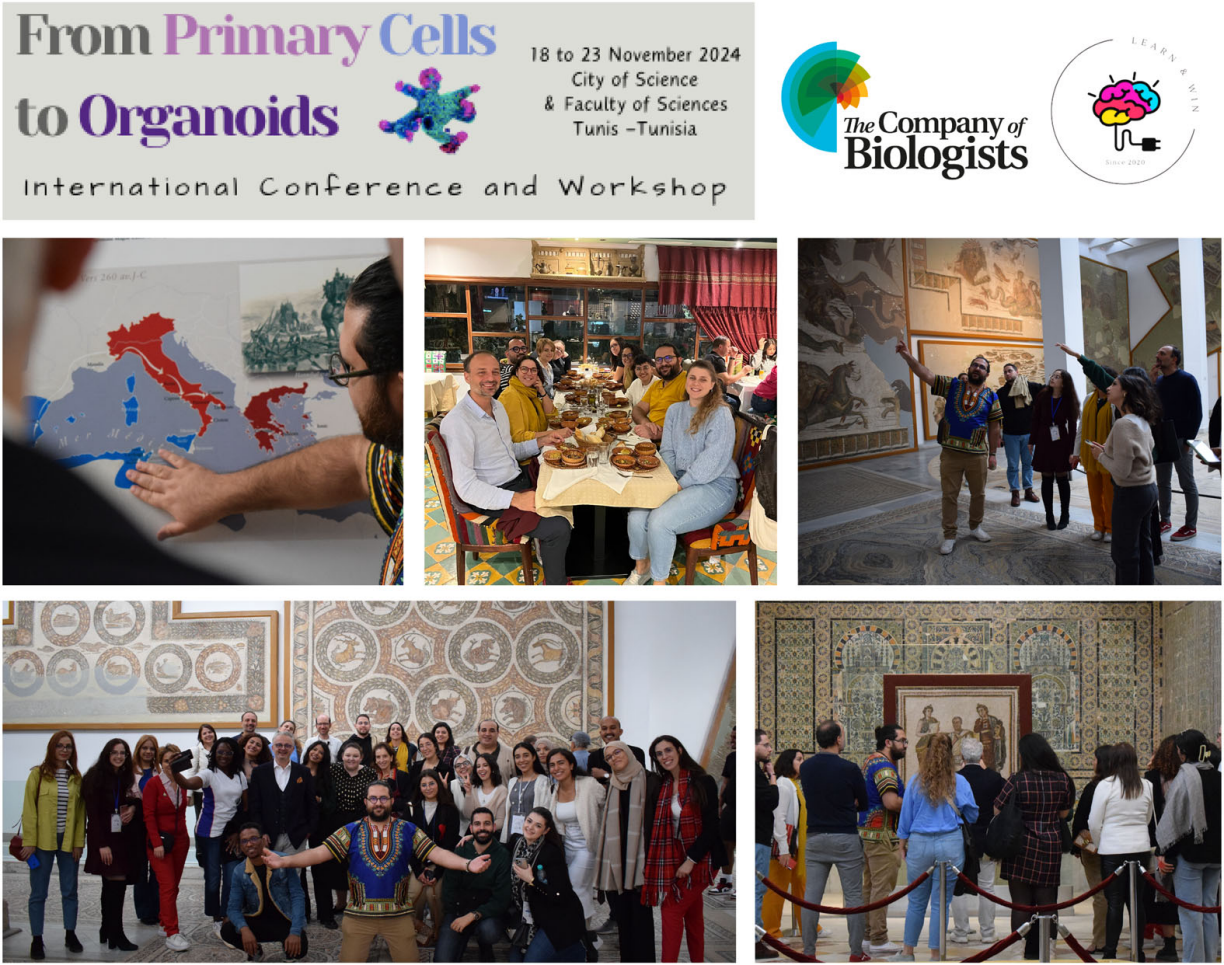
Social events and networking.

The event was truly a success in terms of international participation, as we had colleagues and participants from North Africa (Mauritania, Morocco, Algeria and Egypt), Europe (Turkey, Greece, Italy, UK) and South Africa, so the social event was a must to enhance collaboration and exchange. In addition to the gala dinner for the trainers, speakers and workshop participants, we organised evening excursions for the international guests to enjoy Tunisian street food. We also organised a visit on the last day to the largest museum in Tunisia, the Bardo National Museum, famous for its large collection of mosaics. We created a special guided tour mixing archaeology and life sciences (medicine, agriculture, fishing and other unique pieces of the museum) with prepared storytelling as we had beautiful ancient grandchildren within the visitors, Numidia (for Mauritania, Morocco and Algeria), Carthage (for Tunisia of course), Rome (for Italy), Athena (for Greece), Egypt and Constantinople (for Turkey) (Fig. 4).

## Take-home message

The aim of our event was quite direct: we have to master cell culture in our laboratories and master both classical and complex cell culture protocols. We also have to face and solve bench problems in order to generate valuable data for our research. That is why we invited some of the best researchers in the field to share their tips, tricks and feedback on cell culture in relation to what they do and what they face. In addition, our conference and workshop aimed to highlight the need to move forward and start organoids culture, as they are the future of preclinical assays in biomedicine, and we, as North African institutions and laboratories, need to join the international standards and not remain on the edge of development in science.

In summary, conference provided and supported:

1. **Comprehensive knowledge.** Mastering cell culture techniques from primary cells to organoids provides researchers with a comprehensive understanding of cellular behaviour, enabling more accurate and reproducible experimental results.
2. **Skills development.** Hands-on workshops provided participants with the practical skills to apply the cell culture techniques learned in their own research.
3. **A bridge between basic and translational research.** The evolution from 2D to 3D models, spheroids and organoids reflects the development of cell culture systems that better mimic *in vivo* conditions, bridging the gap between basic research and clinical applications.
4. **Innovation in disease modelling and drug development.** Advanced models such as organoids and 3D cultures provide more physiologically relevant platforms for the study of disease mechanisms, drug screening and personalised medicine, reducing reliance on animal models.
5. **Interdisciplinary collaboration.** Our conference fostered collaboration between scientists, clinicians and industry experts to drive innovation and standardisation in cell culture practices.
6. **Capacity building in North Africa.** The event highlighted the importance of building local expertise in advanced cell culture techniques to enable researchers and institutions in North Africa to contribute to global scientific advances and address regional health challenges.
7. **Connections between regions and focus on regional health priorities.** By bringing together international experts and local researchers, the conference fostered knowledge exchange, collaboration and mentorship, helping to bridge the gap between North Africa and the global scientific community. It also enabled researchers in North Africa to tackle region-specific diseases (e.g. infectious diseases, genetic disorders and cancer) with more relevant and innovative approaches.
8. **Inspiration for the next generation.** By hosting such a high-profile event in North Africa, the conference inspired young scientists and students in the region to pursue careers in cutting-edge biomedical research and biotechnology.

## References

[BIO061970C1] Bourzam, A., Hamdi, Y., Bahdoudi, S., Duraisamy, K., El Mehdi, M., Basille-Dugay, M., Dlimi, O., Kharrat, M., Vejux, A., Lizard, G. et al. (2024). Octadecaneuropeptide, ODN, promotes cell survival against 6-OHDA-induced oxidative stress and apoptosis by modulating the expression of miR-34b, miR-29a, and miR-21in cultured astrocytes. *Cells* 13, 1188. 10.3390/cells13141188 39056770 PMC11487398

[BIO061970C2] Capes-Davis, A., Theodosopoulos, G., Atkin, I., Drexler, H. G., Kohara, A., MacLeod, R. A. F., Masters, J. R., Nakamura, Y., Reid, Y. A., Reddel, R. R. et al. (2010). Check your cultures! A list of cross-contaminated or misidentified cell lines. *Int. J. Cancer* 127, 1-8. 10.1002/ijc.25242 20143388

[BIO061970C3] eBioMedicine. (2022). The 3Rs of animal research. *EBioMedicine* 76, 103900. 10.1016/j.ebiom.2022.103900 35221013 PMC8882996

[BIO061970C4] Jemaa, M., Mokdad Gargouri, R. and Lang, F. (2023). Polo-like kinase inhibitor BI2536 induces eryptosis. *Wien. Med. Wochenschr.* 173, 152-157. 10.1007/s10354-022-00966-7 36178637

[BIO061970C5] Lorsch, J. R., Collins, F. S. and Lippincott-Schwartz, J. (2014). Cell Biology. Fixing problems with cell lines. *Science* 346, 1452-1453. 10.1126/science.1259110 25525228 PMC5101941

[BIO061970C6] Park, G., Rim, Y. A., Sohn, Y., Nam, Y. and Ju, J. H. (2024). Replacing animal testing with stem cell-organoids: advantages and limitations. *Stem Cell Rev. Rep.* 20, 1375-1386. 10.1007/s12015-024-10723-5 38639829 PMC11319430

[BIO061970C7] Simon Serrano, S., Sime, W., Abassi, Y., Daams, R., Massoumi, R. and Jemaa, M. (2020). Inhibition of mitotic kinase Mps1 promotes cell death in neuroblastoma. *Sci. Rep.* 10, 11997. 10.1038/s41598-020-68829-y 32686724 PMC7371706

